# ATP-dependent transporters: emerging players at the crossroads of immunity and metabolism

**DOI:** 10.3389/fimmu.2023.1286696

**Published:** 2023-10-31

**Authors:** Akshaya Balasubramanian, Mark S. Sundrud

**Affiliations:** ^1^ Department of Microbiology and Immunology, Geisel School of Medicine at Dartmouth, Hanover, NH, United States; ^2^ Department of Medicine, Geisel School of Medicine at Dartmouth, Hanover, NH, United States; ^3^ Center for Digestive Health, Dartmouth Health, Lebanon, NH, United States; ^4^ Dartmouth Cancer Center, Lebanon, NH, United States

**Keywords:** ABC transporters, MDR1, P-glycoprotein, metabolism, reactive oxygen species, TCR signaling, oxidative stress, redox

## Abstract

Nearly 50 ATP-binding cassette (ABC) transporters are encoded by mammalian genomes. These transporters are characterized by conserved nucleotide-binding and hydrolysis (i.e., ATPase) domains, and power directional transport of diverse substrate classes – ions, small molecule metabolites, xenobiotics, hydrophobic drugs, and even polypeptides – into or out of cells or subcellular organelles. Although immunological functions of ABC transporters are only beginning to be unraveled, emerging literature suggests these proteins have under-appreciated roles in the development and function of T lymphocytes, including many of the key effector, memory and regulatory subsets that arise during responses to infection, inflammation or cancers. One transporter in particular, MDR1 (Multidrug resistance-1; encoded by the *ABCB1* locus in humans), has taken center stage as a novel player in immune regulation. Although MDR1 remains widely viewed as a simple drug efflux pump in tumor cells, recent evidence suggests that this transporter fills key endogenous roles in enforcing metabolic fitness of activated CD4 and CD8 T cells. Here, we summarize current understanding of the physiological functions of ABC transporters in immune regulation, with a focus on the anti-oxidant functions of MDR1 that may shape both the magnitude and repertoires of antigen-specific effector and memory T cell compartments. While much remains to be learned about the functions of ABC transporters in immunobiology, it is already clear that they represent fertile new ground, both for the definition of novel immunometabolic pathways, and for the discovery of new drug targets that could be leveraged to optimize immune responses to vaccines and cancer immunotherapies.

## 1 Introduction

The field of immunometabolism has exploded over the past two decades. Among many notable discoveries, it is now understood that the activation and differentiation of immune cells – including T cells – involves rapid and profound metabolic reprogramming. On one hand, antigen- and TCR-mediated increases in aerobic glycolysis and mitochondrial respiration allows primed T cells to meet the bioenergetic and biosynthetic demands of cell growth and proliferation. On the other, differences in the magnitude and type of metabolic activities an antigen-primed T cell undertakes can play instructive roles in the commitment of emergent effector, memory and/or regulatory T cell subsets (1–5). CD8 effector T cells and CD4 Th17 and Th1 cells, for example, rely mainly on glycolysis to support rapid proliferation and potent effector functions, whereas CD8 memory cells and CD4 T regulatory (Treg) cells utilize mitochondrial respiration and fatty acid oxidation (FAO) to establish long-term persistence in nutrient-sparse tissues and execute immunosuppressive functions, respectively (3). It is thus not surprising that genetic or environmental perturbations to metabolic pathways have profound influence over the type and quality of T cell responses; further elucidation of the underlying mechanisms holds promises of revealing new preventative or treatment strategies for human infectious or malignant diseases.

Flux through growth-supporting metabolic pathways requires active transport of numerous organic and inorganic molecules across biological membranes. Adenosine triphosphate (ATP)-binding cassette (ABC) transporters constitute one of the largest super-families of transmembrane proteins encoded by the human genome. These transporters utilize ATP hydrolysis to power directional translocation of diverse substrates, against chemical gradients, and across lipid membranes, including the plasma membrane and membranes of intracellular organelles (6). ABC transporters are evolutionarily conserved and present throughout all kingdoms of life, from prokaryotes to humans (7), with major functions centering on either the direct promotion of cellular (e.g., lipid, heme) metabolism, or facilitating cellular detoxification via transport of potentially toxic metabolic byproducts or xenobiotic compounds (8–11). Indeed, loss of function polymorphisms in human ABC transporter loci are now linked to many human diseases, including anemia, obesity, atherosclerosis (AS), congenital cholestasis, peroxisome disorders, cystic fibrosis (CF) and Tangier disease (TD) (8, 9, 12).

More recent studies have begun to highlight direct, important and endogenous functions of ABC transporters in adaptive immune regulation generally, and development and function of T cells specifically (13–25). Here, we discuss the current state of understanding of the ABC transporters in regulating T cell differentiation and function, while also providing forward-looking perspectives as to how transport-dependent cellular metabolic pathways may intersect with antigen receptor signaling to shape T cell lineage commitment, and even the clonality of emergent effector and memory T cell pools.

## 2 Classification and structure of ABC transporters

48 human genes encode ABC transport proteins, most of which have direct orthologs in mice and lower vertebrates. In the early 2000’s, ABC transporters were renamed and reclassified into seven sub-families (ABCA to ABCG), based on phylogenetic analysis and sequence/structural similarity (7, 26–28). Members of the ABCE and ABCF sub-families are notable in that they do not appear to function as transporters per se, but rather participate in translational regulation and mRNA surveillance (29–32).

X-ray crystallographic studies of several ABC transporters have provided atomic-level resolution of the canonical structure of ABC transporters, and specifically the organization of four main functional domains — two nucleotide binding domains (NBD1, NBD2) and two transmembrane domains (TMD1, TMD2). Most eukaryotic ABC transporters are expressed as either a single polypeptide containing all four functional domains, or as half-transporters capable of homo- or hetero-dimerization (33). The NBD contain several conserved functional motifs, whereas the TMDs are more variable and contain 6-11 membrane-spanning α-helices which form the transmembrane pore and mediate substrate binding (33). Diversity amongst TMDs allow the various ABC transporters to bind, and subsequently transport, diverse substrate classes (e.g., heme, lipids, xenobiotics, etc.), and also underlies the phylogenetic relationships between sub-family members. In all cases, ABC transport activity involves ATP-dependent NBD dimerization, which induces conformational changes in the TMDs that exposes the inner region of the pore to the outside and allows for unidirectional transport against chemical gradients (34).

## 3 ABC transporters in T cells

The first described and arguably most famous ABC transporters within the immune system are the transporters associated with antigen processing (TAP1/2), which transport cytosolic peptides into the endoplasmic reticulum (ER) for loading onto MHC Class I molecules (35). Another immunologically notable ABC transporter is ABCC7 [*a.k.a*., cystic fibrosis transmembrane conductance regulator (CFTR)], which functions as an apical chloride channel on lung epithelium, and whose loss-of-function leads to chronic bacterial infections of the lung (36). However, numerous ABC transporters are now recognized for filling essential functions within the adaptive immune system generally, and immunometabolism specifically. The obligate mitochondrial transporters ABCB7 and ABCB10, for example, are considered heme transporters based on their roles in erythropoiesis, and are necessary for the development of B cells and CD4 memory T cells, respectively (23, 24). In addition, lipid and multi-drug transporters have recently emerged as key regulators of T cell metabolism and function (**Table 1**).

**Table 1:** ABC transporters and their associated function in T cells.

ABC Transporter (in humans)	Mouse ortholog	Transport Substrate	Function in T cells	References
*ABCA1*	*Abca1*	Phospholipid and cholesterol	Regulates TCR signaling by maintaining lipid raft composition in peripheral lymphocytes.	Armstrong et al., (13); Zhao et al., (25); Bazioti et al., (37)
*ABCA7*	*Abca7*	Phospholipid	Disrupts lipid rafts and CD1d expression in iNKT cells.	Nowyhed et al., (20)
*ABCB1*	*Abcb1a*	Broad-spectrum drug efflux pump	Suppresses oxidative stress and promotes survival of T cells found in mucosal sites and CD8 T cells responding to infection.	Boddupalli et al., (16); Cao et al., (18); Xie et al., (21); Chen et al., (22)
	*Abcb1b*			
*ABCB10*	*Abcb10*	Possible heme transporter	Necessary to maintain stable CD4 memory pool and aid in switching metabolic states during activation.	Sun et al., (24)
*ABCC4*	*Abcc4*	Endogenous metabolites and xenobiotics	Induced in response to hypoxic conditions in Th17 cells.	Xie et al., (21)
*ABCG1*	*Abcg1*	Phospholipid and cholesterol	Regulates TCR signaling by maintaining lipid raft composition in thymic and peripheral lymphocytes.	Bensinger et al., (38); Armstrong et al., (13); Cheng et al., (17); Bazioti et al., (37)
*ABCG2*	*Abcg2*	Broad-spectrum drug efflux pump	Characteristic features of Trm cells and is necessary for maintenance of Trm niche in the gut.	Boddupalli et al., (16)
	*Abcg3*			

### 3.1 Lipid transporters

As many as 20 human ABC transporters are thought to transport discrete lipids or lipid metabolites. Among these, ABCA1 and ABCG1 are perhaps the best characterized for the roles they play in cholesterol transport and homeostasis, and for regulating TCR signaling intensity (13, 17, 25, 37). These transporters maintain structural organization and integrity of lipid bilayers, at least in part by regulating the number and density of cholesterol-rich lipid rafts (13, 17, 25). Given the key and ubiquitous roles of lipid rafts in signal transduction generally, and in immune cell signaling specifically, it is not surprising that these cholesterol and lipid transporters exert powerful influences over the development and function of T cells (39).

**Figure 1:**
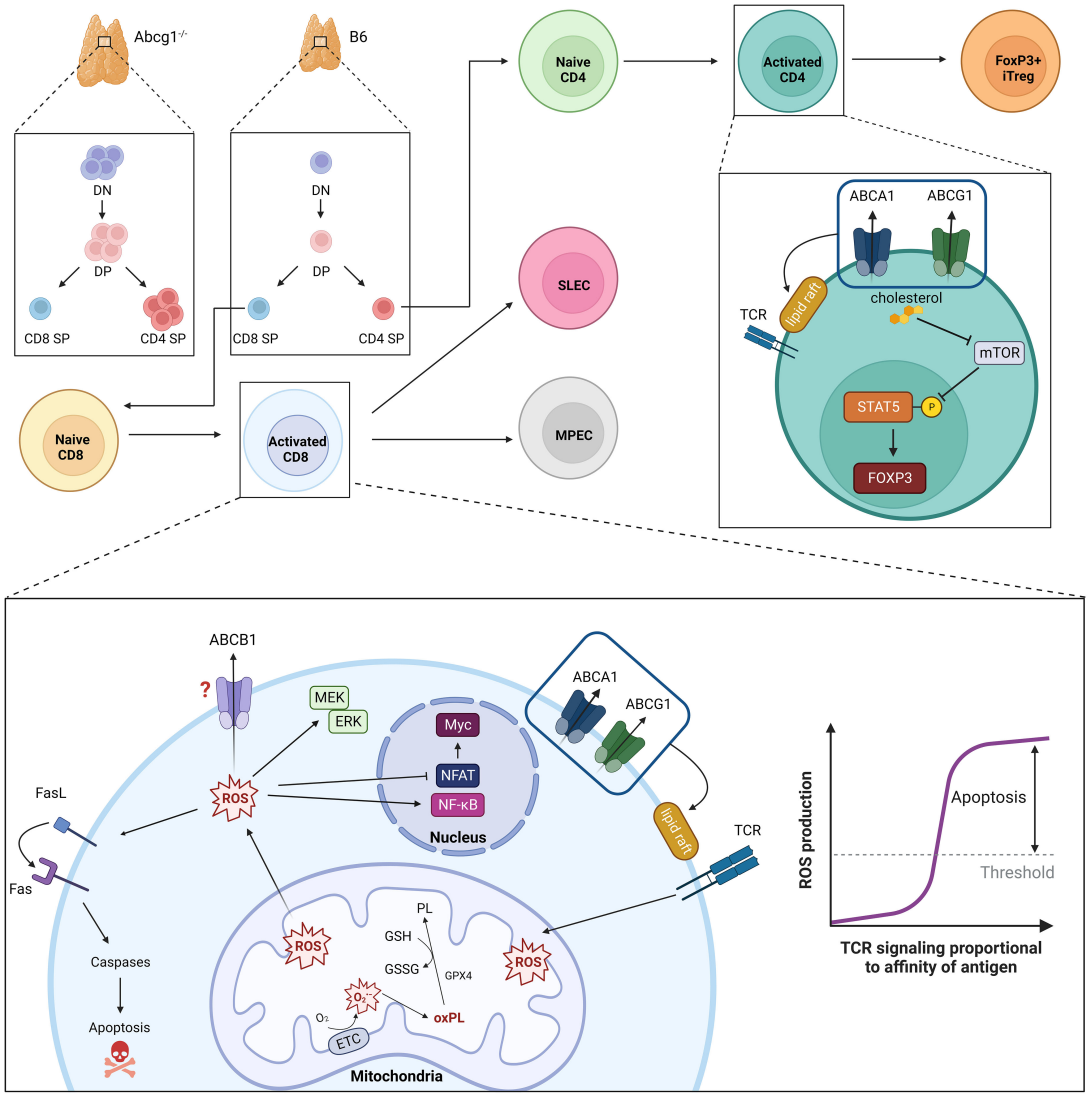
ABC transporters in regulation of T cell development, TCR intensity and oxidative stress. Loss of ABCG1 transport activity results in increased thymic cellularity and frequency of double negative (DN), double positive (DP) and CD4 single positive (SP) cells. Intensity of TCR signaling is modulated by ABCA1- and ABCG1-mediated phospholipid transport; loss of these transporters is associated with increased numbers and densities of cholesterol-rich lipid rafts. Parallel accumulation of intracellular cholesterol also promotes peripheral development of CD4+Foxp3+ induced (i)Treg cells. TCR signaling strength and duration is proportional to the amount of intracellular ROS produced. Moderate ROS levels (i.e., during T cell responses to low- or intermediate-affinity antigens) facilitate T cell proliferation, differentiation and survival through MEK-ERK1/2 and NF-κB activation. Conversely, elevated ROS levels (i.e., produced during T cell responses to high-affinity ligands) impair NFAT and Myc expression and promote activation-induced cell death (AICD) via Fas-Fas ligand (FasL). To maintain functional, but not toxic, ROS levels, activated T cells leverage endogenous anti-oxidant systems (e.g., glutathione peroxidase, GPX4) to reduce oxidized phospholipids (oxPL) within mitochondrial membranes using reduced glutathione (GSH) as a cofactor. MDR1 also suppresses oxidative stress in activated T cells, though underlying mechanisms remain ill-defined.

*Abcg1^-/-^* mice display larger thymuses than wild-type controls due to thymocyte hyperproliferation and an increase in total thymic cellularity (13). ABCG1 also affects thymic and peripheral development of Foxp3^+^ Treg cells (17). An increase in thymic (i.e., ‘natural’) Treg cells is observed when *Abcg1* is conditionally ablated in T cells using *Lck-Cre* (17). In the periphery, preferential differentiation of naïve CD4 T cells into Tregs is evoked in the absence of ABCG1 by an increase in cellular cholesterol levels, which inhibits mammalian target of rapamycin (mTOR), triggers the phosphorylation and activation of signal transducer and activator of transcription (STAT)-5, and promotes the induction and stability of *Foxp3* expression (**Figure 1**) (17).

ABCA1 and ABCG1 are necessary for the maintenance of peripheral T cell homeostasis (13, 17, 25, 37). Expression of *Abca1* and *Abcg1* decreases following naïve T cell activation, which in turn promotes increased membrane cholesterol content to support cell growth and proliferation (38, 40). Intriguingly, ABCA1 and ABCG1 appear to work synergistically in T cells, as ablation of both *Abca1* and *Abcg1* in a T-cell specific manner enhances TCR signaling in CD4 and CD8 T cells (25, 37). This enhancement of TCR signaling has been attributed to an increase in lipid raft formation (13). On the other hand, upregulation of *Abcg1* expression in T cells by the nuclear receptor, liver X receptor (LXR), reduces T cell proliferation (38). The need for tightly regulated lipid transport to support TCR signaling is not limited to conventional T cells; ABCG1, as well as another lipid transporter, ABCA7, are both essential for thymic maturation of invariant natural killer T (iNKT) cells (14, 20). Hence, regulation of cholesterol efflux by ABCA1, ABCG1 and ABCA7 each appear to participate in the regulation of signal transduction, and in establishing a full complement of peripheral lymphocytes.

### 3.2 Multi-drug transporters

Another class of ABC transporters recently implicated in immune regulation are the so-called multi-drug transporters. This historical classification derives from a combination of functional studies in eukaryotic cells and examination of homologs in bacterial model systems, which together show that multi-drug transporters are capable of effluxing a variety of structurally-unrelated cytostatic drugs from tumor cells (18). However, considering the repeated and high-profile failures of drugs designed to block multi-drug transporters in clinical cancer studies (41), and the emerging endogenous functions of at least some of these transporters, this semantic classification may require updating. Indeed, ‘multi-drug’ transporters – including multidrug resistance protein 1 (MDR1/ABCB1), multidrug resistance protein 4 (MRP4/ABCC4) and breast cancer resistance protein (BCRP1/ABCG2) – each display considerable evolutionary conservation and thus may also be considered ‘orphan’ transporters, as endogenous transport substrates have not yet been described. An important and common theme among these multi-drug transporters is their incredibly broad substrate specificity; MDR1, for example, has been proposed to efflux a variety of synthetic drugs, antiretroviral drugs, glucocorticoids, lipids, fluorescent dyes and even peptide hormones (in yeast) (42–45). Yet many of these same transporters display endogenous antioxidant functions in several cell types and tissues during normal physiology, including in T cells. Thus, it is worth considering that the core evolutionary function of ‘multi-drug’ transporters centers on the regulation of oxidation-reduction (redox) metabolism, which have become masked in recent decades by their proclivity to efflux 20^th^ century medicines.

MDR1 is the most extensively characterized multi-drug transporter within the ABC family for its role as a regulator of oxidative stress. MDR1 is endogenously expressed in a variety of normal cell types and tissues, with highest levels seen in the liver, intestines, brain, kidney and adrenal glands (46–48). In the hematopoietic system, comprehensive profiling of a fluorescent *Abcb1a*-ametrine reporter allele in mice showed that MDR1 is expressed in a number of mature innate and adaptive lymphocytes, but is notably absent in most hematopoietic stem and progenitor cells, early thymocytes, and also mature granulocyte and B lymphocyte lineages (22). In CD4 T cells, MDR1 expression is absent in naïve cells, low in Foxp3^+^ Treg cells, but increased markedly in pro-inflammatory effector subsets, namely IFNγ-secreting Th1 and IL-17-producing Th17 cells, in particular following infiltration of small intestine lamina propria (15). In contrast to this conditional expression in CD4 T cells, MDR1 expression is both constitutive and developmentally regulated in cytotoxic lymphocyte lineages (e.g., NK cells, iNKT cells, CD8 T cells) where the expression of MDR1 is at least partly controlled by Runx family transcription factors (22). Expression of MDR1 (and ABCG2) are also characteristic of tissue-resident memory (Trm) CD8 T cells, as well as of human mucosal associated invariant T (MAIT) cells (16, 19, 49). In line with constitutively high MDR1 expression, mouse CD8 T cells null for MDR1 transport activity (*Abcb1a/b^-^* ^/-^) are incapable of becoming productively activated, of accumulating efficiently in response to acute viral or bacterial infections, and of forming functional memory cells (22). By contrast, naïve CD8 T cells do not require MDR1 function for steady-state persistence (22), which suggests that MDR1 transport activity in CD8 T cells is primarily called upon after TCR-stimulation. Indeed, the inability of *Abcb1a/b^-^* ^/-^ CD8 T cells to accumulate following TCR-stimulation is due to increased cell death, not reduced proliferation, and coincides with a failure of these cells to suppress oxidative stress and maintain functional mitochondria (**Figure 1**) (22). Others have shown that Th17 cells require both MDR1 and ABCC4 to suppress oxidative stress during states of hypoxia (21). Further, high MDR1 expression in Th1 and Th17 cells in the distal small intestine (i.e., ileum) counters oxidative stress induced by naturally circulating bile acids, an abundant class of liver-derived lipid-emulsifying metabolites that are also potent oxidizing agents (18, 50). Even outside of the immune system, MDR1 has been shown to suppress oxidative stress and safeguard mitochondrial integrity in colonic epithelial cells (51). Collectively, these findings support a broader and more fundamental role for the MDR1 transporter in suppressing oxidative stress upon T cell exposure to intra- or extra-cellular oxidizing agents (e.g., toxic metabolic byproducts, bile acids, etc.). Such transport-dependent antioxidant functions of MDR1 raise intriguing new concepts in the convergence of TCR signaling, metabolic reprogramming, oxidative stress, and even clonal selection in T cells.

## 4 Determinants of peripheral T cell responses: TCR signaling and ROS

T cell proliferation, lineage commitment, and the execution of effector or regulatory functions are all markedly influenced by the strength and duration of TCR/peptide-MHC engagement (52–54). Upon infection, the size of the antigen-specific CD8 T cell compartment correlates directly with the duration of antigen exposure (55). Strength and duration of TCR signaling also shapes both CD4 and CD8 memory T cell compartments, as highlighted by the preferential skewing of the memory pool towards clones with higher-affinity TCRs (55–57). It is increasingly clear that TCR signaling thresholds also regulate functional outcomes of individual T cell clones (e.g., proliferative capacity, differentiation, etc.); pathways that regulate successful integration of TCR, co-stimulatory and cytokine signaling pathways thus determine the magnitude, type and antigen-specificities of T cell responses.

Proximal TCR signaling induces rapid Ca^2+^ release into the cytosol from reserves in the endoplasmic reticulum (ER), which upon being emptied, activate extracellular Ca^2+^ influx across the plasma membrane (58, 59). Besides serving as a potent signal to activate early transcription factors, such as the NFATs, cytosolic Ca^2+^ is also taken up in mass by mitochondria, which facilitates ATP synthesis and promotes corresponding increases in levels of reactive oxygen species (ROS) (60). This increase in intracellular ROS occurs within minutes of TCR activation, with the majority of ROS production in activated T cells owing to mitochondrial superoxide (O_2_^•−^) that “leak” from complexes I and III of the mitochondrial electron transport chain (ETC) (61).

ROS function as key secondary messengers in T cells and are required, at moderate levels, to promote proliferation, differentiation and survival. The activation of extracellular signal-regulated kinase (ERK) 1/2, for example, is highly ROS-dependent, and promotes activation and translocation of several transcription factors, including AP-1 family members, that are necessary for T cell growth and proliferation (62, 63). ROS production has also been implicated in promoting IL-2 production in activated T cells through NF-κB activation (64). At the same time, inappropriately elevated ROS levels are highly cytotoxic, and can stimulate apoptosis by covalently modifying and damaging proteins, nucleic acids, and lipids (65). Oxidative stress also promotes increased expression of Fas ligand (FasL), which on binding to Fas initiates the recruitment and activation of caspases causing activation induced cell death (AICD) (**Figure 1**) (62, 66, 67).

Considering the dual nature of intracellular ROS – where ROS is both required for T cell activation, but also toxic at increased levels – activated T cells upregulate a host of anti-oxidant enzymes (superoxide dismutases (SODs), catalases, glutathione peroxidases (GPXs), glutathione reductases, etc.) to convert ROS into less reactive products, or to increase production of ROS scavenging molecules, such as glutathione, ascorbate, pyruvate, α-ketoglutarate, and oxaloacetate (65). Disruptions in these anti-oxidant systems result in elevated ROS production and affect metabolic reprogramming in developing T cells. Inhibition of glutathione production, which is important for ROS buffering, negatively affects TCR-induced Myc expression and NFAT activation, which are essential for activated CD8 T cells to switch to glycolytic metabolism (68). Similarly, loss of *Gpx4* impairs CD8 T cell responses to viral infections due to accumulation of lipid peroxides, leading to death by ferroptosis (69). Thus, productive immune responses demand that T cells tightly regulate both the production and scavenging of intracellular ROS. Discrete roles for ABC transporters in T cell redox regulation and metabolism remain unclear, but stand to provide exciting new molecular insights into the formation and regulation of effector and memory T cells.

## 5 Conclusions and future perspectives

Considering that both the developmental trajectories and functions of individual T cell clones involve unique TCR signaling dynamics, and thus discrete redox demands, it becomes increasingly important to decipher whether and how ABC transporters not only regulate the magnitude of monoclonal (i.e., TCR transgenic) T cell responses, but also the selection of T cell clones specific for high- vs. low-affinity antigens. Given the preponderance of available evidence, it is reasonable to expect that MDR1, and perhaps other lipid and multidrug transporters, preferentially regulate T cell responses to abundant and high-affinity (i.e., immunodominant) antigens, in which the highest levels of intracellular ROS are generated. If true, these pathways (and ABC transporters) may prove vital for advancing the next generation of medicines that can improve T cell-mediated immunity to infectious diseases and cancers, where the survival of T cells specific for immunodominant antigens are paramount for efficacy.

Despite being one of the largest families of human transmembrane proteins, a comprehensive understanding of endogenous ABC transporter functions remains lacking. Historically, this has been due to the inherent difficulty of working with membrane proteins and transporters, as well as a dearth of contemporary research reagents (e.g., engineered mouse alleles, antibodies, etc.). While significant strides have been made in recent decades with regard to the structural organization of these transporters, meaningful insights into the physiological and immunological functions of these transporters have lagged. Mechanistic understanding of these transporters remains largely based on data from pharmacological and *in vitro* studies, but it is becoming increasingly apparent that these transporters have important and context-dependent functions that need to be evaluated *in vivo*, during both normal- and patho-physiology. This is especially true for transporters, like MDR1, which remain classified as ‘multi-drug’ efflux pumps, despite continued elaboration of new and potent endogenous functions in *in vivo* mouse and *ex vivo* human systems. For these, the key unanswered question is why would an ABC transporter be conserved throughout evolution unless it serves a core endogenous function? The continued pursuit of basic science, and the advancement of next-generation medicines, await answers.

## Author contributions

MS: Conceptualization, Funding acquisition, Supervision, Writing – review & editing. AB: Conceptualization, Writing – original draft, Writing – review & editing.
